# Severe Acute Hepatitis B in HBV-Vaccinated Partner of a Patient with Multiple Myeloma Treated with Cyclophosphamide, Bortezomib, and Dexamethasone and Autologous Stem Cell Transplant

**DOI:** 10.1155/2017/2463953

**Published:** 2017-03-27

**Authors:** Majed M. Almaghrabi, Kyle J. Fortinsky, David Wong

**Affiliations:** ^1^Division of Internal Medicine, University of Toronto, Toronto, ON, Canada; ^2^Division of Hepatology, University of Toronto, Toronto, ON, Canada

## Abstract

Hepatitis B reactivation can occur with various forms of immunosuppression. Cyclophosphamide, Bortezomib, and Dexamethasone (CYBOR-D) chemotherapy is commonly used for the treatment of multiple myeloma and has not been noted in guidelines to be causative in HBV reactivation. Indeed, current guidelines do not recommend providing antiviral prophylaxis to patients with prior HBV infection. We present a case of HBV reactivation as a result of CYBOR-D and autologous stem cell transplant which is complicated by the patient's partner who developed acute hepatitis B. Our case highlights the need to review the role of antiviral prophylaxis for patients undergoing treatment of multiple myeloma and also the role of ensuring immunity for close contacts of these patients who may also be at risk.

## 1. Introduction

The natural history of chronic hepatitis B virus (HBV) infection is characterized by various stages that are influenced by the host immune response. Resolved HBV infection is defined by undetectable levels of HBV proteins (HBsAg) and viral load (HBV DNA) in a patient with evidence of previous exposure (positive anti-HBc) [1, 2]. HBV reactivation occurs most often in patients with resolved HBV infection who are exposed to profound immunosuppression. Current guidelines describe certain high-risk patients who may benefit from antiviral therapy as prophylaxis against HBV reactivation including those with certain forms of cancer or those on specific chemotherapeutic agents [3, 4]. However, the guideline recommendations are largely based upon expert opinion.

Multiple myeloma (MM) is a cancer resulting in monoclonal expansion of IgG against one epitope potentially at the expense of immunity to other antigens such as HBV. While the precise mechanism within the host immune response is largely unknown, recent studies suggest that T-cells may not be the major players in the natural history of chronic HBV infection [5]. Importantly, since therapies targeted against CD20 can result in HBV reactivation even after HBsAg loss, this suggests that B-lymphocytes and plasma cells likely play a role in immunity against HBV infection [6]. In patients undergoing autologous stem cell transplant (ASCT), host immunity is presumably restored immediately after chemoablation of cancer cells [7]. Moreover, HBV immunity can be adoptively transferred in allogeneic bone marrow transplants. For example, the marrow from a donor who has immunity to HBV can result in HBsAg clearance in a recipient with chronic HBV infection [8]. Conversely, marrow from a donor without immunity to HBV can result in HBsAg recurrence in a recipient who had prior HBV infection that resolved [9].

A few recent studies have reported cases of HBV reactivation in patients with MM being treated with Bortezomib-based chemotherapy [10, 11]. Tsukune et al. reported 9 patients with MM who received Bortezomib-based chemotherapy and developed HBV reactivation [12]. Lee et al. reported 12 patents with MM who all received ASCT (2 of whom were on Bortezomib therapy) and all developed HBV reactivation [13].

The HBV serological status and the type of immunosuppressive therapy are the major determinants in the current guidelines for prevention and treatment of HBV in patients undergoing immunosuppressive therapy. Patients who are HBsAg positive/anti-HBc positive or HBsAg negative/anti-HBc positive undergoing immunosuppressive therapy with B-cell depleting agents, anthracycline derivatives, tyrosine kinase inhibitors, cytokine inhibitors, or integrin inhibitors are recommended to receive antiviral prophylaxis as they are considered to be at moderate to high risk of HBV reactivation [3]. Proteosome inhibitors (e.g., Bortezomib) are not mentioned in the guidelines which suggests they do not require antiviral prophylaxis. Neither the product monograph for Bortezomib nor FDA safety labeling mentions a risk of hepatitis B reactivation. Additionally, current guidelines do not mention screening family members or household contacts for immunity against HBV even when patients themselves are at high risk of HBV reactivation.

The current report describes a case whereby HBV immunity was maintained after the diagnosis of multiple myeloma but was subsequently lost after chemotherapy and autologous stem cell transplant. The current case report adds to the increasing body of literature that suggests Bortezomib-based chemotherapy regimens may lead to HBV reactivation. Importantly, our case is the first report published on acute hepatitis B in a family member of a patient who develops HBV reactivation despite a history of HBV vaccination. Our case highlights important considerations in the management of patients who are undergoing immunosuppressive therapy and their families.

## 2. Case  1

A 68-year-old Canadian man, originally born in Italy, was referred to our hepatology clinic for management of HBV reactivation after his wife was diagnosed with acute HBV (see Case 2 for details of wife). He was diagnosed with MM and was treated with five cycles of Cyclophosphamide, Bortezomib, and Dexamethasone (CYBOR-D) prior to undergoing an autologous stem cell transplant (ASCT). After the transplant, he was put on Lenalidomide for maintenance therapy. Prophylactic HBV antiviral therapy was not given. His past medical history was remarkable only for resolved HBV infection (HBsAg negative, anti-HBc positive, and anti-HBs positive), which was noted prior to transplantation.

After CYBOR-D chemotherapy, anti-HBs became negative and ALT was elevated. In February 2013 his anti-HBs was positive and in December 2014 his anti-HBs was found to be negative. In February 2016, his family physician provided HBV vaccination presumably because he was unaware of his prior HBV infection. Testing for HBV reactivation was not done until his wife presented with transaminitis, jaundice, and fatigue.

**Figure 1:**
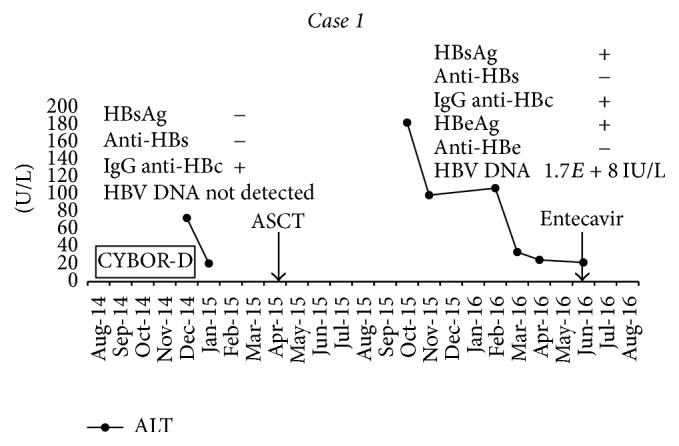
Liver enzymes and HBV tests in relation to chemotherapy, autologous stem cell transplant, and entecavir therapy.

Once his wife was diagnosed with acute HBV, he was diagnosed with HBV reactivation: HBsAg positive (titer > 124,925 IU/mL), HBeAg positive, HBV DNA > 1.70*E*8 IU/mL, and ALT persistently normal. His anti-HCV was negative and he remained immune to hepatitis A. In retrospect, liver enzymes had been abnormal (ALT peak 182) during the HBV vaccination period but were normal when his wife presented with acute hepatitis (see Figure 1). An abdominal ultrasound was unremarkable and a FibroScan was consistent with F1 (minimal) fibrosis. He was started immediately on entecavir for HBV reactivation.

At 4 months' follow-up, he remained asymptomatic from a liver perspective. His liver enzymes and liver function were normal and his HBV viral load was reduced to 4.64*E*4 IU/mL on entecavir.

## 3. Case  2

A 68-year-old Canadian woman, originally born in Canada, was admitted to hospital for 2 days after developing jaundice and severe fatigue over the past two weeks. Her past medical history was significant for osteoarthritis, where she was taking daily acetaminophen (<2 grams per day). She denied any sick contacts, recent travel, or symptoms suggestive of an underlying autoimmune disorder. She denied taking any additional medications or herbal supplements. She was a nonsmoker and denied any alcohol or illicit drug use. She had no prior blood transfusions, tattoos, needle stick injuries, incarceration, or recent travel to an HBV endemic country. She reported receiving prior immunization to hepatitis B in 1988 prior to a trip to India but had not been tested for adequate anti-HBs levels.

On examination, she was afebrile and hemodynamically stable. She appeared jaundiced but there were no signs of hepatic encephalopathy. Liver edge was mildly tender and palpable, 4 cm below the costal margin. There was no splenomegaly, ascites, or other stigmata of chronic liver disease. She had no rashes or arthritis. The remainder of her physical examination was unrevealing.

**Figure 2:**
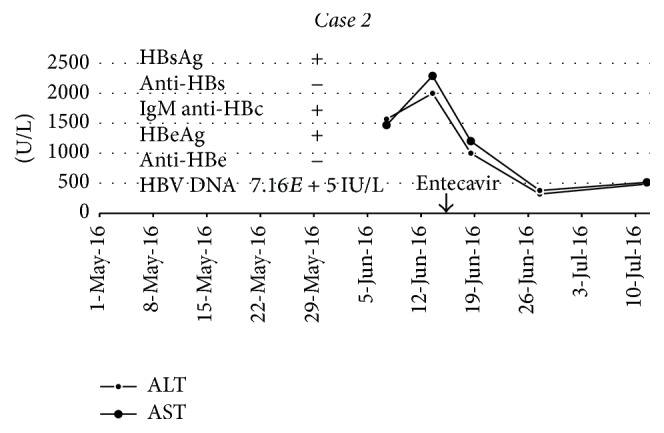
Liver enzymes and HBV tests in relation to entecavir therapy.

Blood tests showed marked transaminitis (see Figure 2), INR 1.3, and Bilirubin up to 411 umol/L. HBV serology was consistent with an acute HBV infection as evidenced by a positive IgM anti-HBc, HBsAg, and HBeAg. Abdominal ultrasound revealed an enlarged liver that was 14.8 cm but was otherwise unremarkable. She was treated with entecavir for symptomatic acute HBV infection and her fatigue mostly resolved after a week of therapy.

At 4 months' follow-up, the patient was feeling well, and her liver enzymes and liver function were normal. Her viral load was undetectable and her HBsAg was negative indicating successfully cleared infection; entecavir therapy was stopped.

## 4. Discussion

Most cases of HBV reactivation in the literature describe HBsAg seroreversion that leads to either low-level viremia without ALT elevation or more significant reactivation with high-level viremia and ongoing immune-mediated liver injury with ALT elevation. Interestingly, our patient in Case 1 developed HBsAg seroreversion with only transient hepatitis before entering what looked like the immune tolerant phase. There have only been 2 previous reported cases of patients with MM who received Bortezomib and had HBV reactivation followed directly by an immunotolerant phase [10, 11].

The precise role of antiviral prophylaxis in patients with resolved HBV who are undergoing ASCT remains controversial [3, 4, 13]. Tsukune et al. recommend serial monitoring of HBsAg or HBV DNA as a strategy to detect reactivation [12]. There do appear to be certain risk factors for HBV. Patients with negative anti-HBs seem to be at higher risk of HBV reactivation when compared to patients with a positive anti-HBs [13].

It is interesting to note that the patient's wife developed acute hepatitis B several months after we suspect the patient developed reactivation. Unfortunately, despite a rise in our patient's liver enzymes after ASCT, he was not recognized to have reactivation until his wife developed acute HBV. The delay between reactivation and his wife's acute HBV infection may be attributable to a rising HBV DNA level during that period as he was becoming more infectious.

There may have been several factors contributing to our patients HBV reactivation including Cyclophosphamide, Bortezomib, Dexamethasone, ASCT, and Lenalidomide therapy. Lenalidomide is a potent immunomodulator and T-cell stimulator. It has been recommended to be used with caution in patients infected with HBV, though the role of immunomodulators in HBV reactivation is still undetermined [14]. ASCT has also been raised as a risk factor for HBV reactivation [15]. Bortezomib, a proteasome inhibitor, has been previously reported in a few cases of HBV reactivation in MM patients [10, 11]. Given its mechanism of action, Bortezomib may impair intracellular viral antigen processing leading to impaired maintenance of T-cell memory [16]. Recent guidelines, however, do not acknowledge that HBV reactivation might occur in patients exposed to Bortezomib therapy [3, 4]. Lastly, there is evidence that systemic corticosteroids such as Dexamethasone may also increase the risk of reactivation [17].

Interestingly, our patient lost anti-HBs, which prompted his family physician to repeat vaccination instead of testing for HBV reactivation. There are no data to support the strategy of HBV vaccination in those who lose anti-HBs after immunosuppression. Although patients with a negative anti-HBs may be at higher risk for reactivation compared to patients with a positive anti-HBs, current guidelines do not suggest prophylaxis for patients based upon their anti-HBs status alone. In general, prophylaxis with HBV antiviral therapy is initiated at the beginning of chemotherapy until approximately 6 months after completing therapy. Moreover, current guidelines do not recommend routine testing for family members or close contacts of patients at risk for reactivation.

The majority of patients with acute hepatitis B recover spontaneously without need for antiviral treatment. However, those who have symptomatic acute HBV infection may benefit from antiviral therapy to shorten duration and severity of symptoms. Those who remain HBeAg positive with high-level viremia more than 3 months after infection may benefit from antiviral therapy to prevent establishment of chronic infection. In regard to treatment, there are different choices of antiviral therapies including lamivudine, tenofovir, and entecavir [18].

This report highlights a case of HBV reactivation in a patient with MM treated with Bortezomib containing chemotherapy. It is still unclear if antiviral therapy versus careful monitoring is the best strategy for such patients. As the risk of reactivation is low and can take place over many years [12], it may be reasonable to monitor HBsAg every 6 months for 3 to 5 years after therapy rather than treating all with antivirals for 5 years.

Our case described a unique situation where the patient lost anti-HBs and vaccination did not prevent reactivation. Clinicians should consider more careful monitoring of patients undergoing Bortezomib-based chemotherapy and pay especially close attention to patients without anti-HBs. HBV vaccination in those who lose anti-HBs should not be undertaken until HBsAg testing to rule out HBV reactivation has been done. Furthermore, testing to monitor for HBV reactivation should be performed routinely during and immediately after treatment. Lastly, our case describes an unusual complication of a family member developing acute HBV as a result of reactivation in her husband. This occurred despite the wife being vaccinated many years priorly. The authors suggest that clinicians consider screening close household contacts of patients at risk for HBV reactivation in order to identify those without immunity to HBV who could benefit from repeat vaccination. Our cases portray the importance of considering both the patient and family members or close contacts who can also be adversely affected by medical treatments.
